# Dual targeting of GSK3B and HDACs reduces tumor growth and improves survival in an ovarian cancer mouse model

**DOI:** 10.1016/j.ygyno.2020.07.005

**Published:** 2020-07-19

**Authors:** Enes Taylan, Fouzia Zayou, Ramachandran Murali, Beth Y. Karlan, Stephen J. Pandol, Mouad Edderkaoui, Sandra Orsulic

**Affiliations:** a Department of Obstetrics and Gynecology, David Geffen School of Medicine, University of California Los Angeles, Los Angeles, CA, USA; b Departments of Medicine, Biomedical Sciences, Radiation Oncology and Surgery, Samuel Oschin Comprehensive Cancer Institute, Cedars-Sinai Medical Center, Los Angeles, CA, USA; c Jonsson Comprehensive Cancer Center, University of California Los Angeles, Los Angeles, CA, USA; d Department of Pediatrics, David Geffen School of Medicine, University of California Los Angeles, Los Angeles, CA, USA

**Keywords:** Ovarian cancer, Histone deacetylase inhibitor, Glycogen synthase kinase 3 beta inhibitor, Chemotherapy resistance, HDAC, GSK3B

## Abstract

**Objective.:**

To investigate the anti-tumor effect of a newly-developed dual inhibitor (APCS-540) of glycogen synthase kinase 3 beta (GSK3B) and histone deacetylases (HDACs) in ovarian cancer cells.

**Methods.:**

The effects of APCS-540 on cancer cell proliferation, migration, invasion and cancer stemness were investigated *in vitro* in human (KURAMOCHI, OVCA420, OVSAHO) and mouse (BR-Luc, ID8, MOSE-HRas-Myc) ovarian cancer cells. Cisplatin-sensitive (A2780) and cisplatin-resistant (A2780cis) cell lines were used to evaluate APCS-540’s effect on chemoresistance. The immunocompetent syngeneic mouse model BR-Luc was used to test the effect of APCS-540 on ovarian cancer progression and survival.

**Results.:**

APCS-540 showed significant anti-tumor effects *in vitro* in both human and mouse ovarian cancer cells. Importantly, APCS-540 demonstrated marked cytotoxicity against cisplatin-resistant cancer cells and reversed cisplatin-resistance when used in combination with platinum. APCS-540 significantly decreased cancer cell invasion. A significant 66% increase in survival was observed in mice treated with APCS-540 compared to control mice.

**Conclusion.:**

Dual inhibition of GSK3B and HDACs *via* APCS-540 showed potent anti-tumor activity *in vitro* and *in vivo*, suggesting that APCS-540 may provide a novel treatment option for ovarian cancer, including the platinum-resistant disease.

## 1. Introduction

Ovarian cancer is the deadliest gynecologic malignancy with approximately 22,000 new cases and more than 14,000 deaths each year in the United States [1]. With no effective means for early detection and no specific symptoms, the vast majority of women with ovarian cancer present with widely disseminated metastatic disease at diagnosis. Despite the work of committed physicians and scientists, the overall survival rate for the most common type of ovarian cancer, high-grade serous ovarian carcinoma, has not significantly improved in over 40 years and remains at ~30%. Standard treatment for ovarian cancer includes cytoreductive surgery coupled with systemic chemotherapy to eliminate any remaining tumor cells and achieve clinical remission. Although remission can be achieved in about 80% of patients with high grade serous ovarian carcinoma, most of these patients experience recurrence due to the presence of chemotherapy-resistant clones of cancer cells. Recent clinical trials with various checkpoint inhibitors alone or in combination with standard therapy for ovarian cancer have been unsuccessful [2]. Thus, effective alternative therapies are urgently needed.

A promising approach to the development of more effective treatments for ovarian cancer is targeting multiple cellular pathways simultaneously to prevent the development of drug resistance in cancer cells, which is the major challenge for standard chemotherapy. Studies have shown that epigenetic dysregulation and alterations in metabolic pathways are crucial components in the development and progression of several solid tumors, including ovarian cancer [3,4]. Glycogen synthase kinase-3 beta (GSK3B) is a glycogen metabolism enzyme that upregulates NF-κB activity, a key driver of proliferation and survival in ovarian cancer cells, and inhibition of GSK3B caused tumor shrinkage in mice [5–7]. Recent studies also showed that inhibition of histone deacetylases (HDACs) effectively suppressed ovarian cancer growth and metastasis by inhibiting expression of PAX8, a critical oncogene in ovarian cancer [8]. Therefore, we hypothesized that dual targeting of metabolic (GSK3B) and epigenetic (HDACs) pathways might significantly improve anti-tumor treatment and overcome chemoresistance in ovarian cancer.

We have previously reported the design, synthesis, and testing of a novel dual GSK3B and HDACs inhibitor Metavert for the treatment of pancreatic adenocarcinoma [9]. In this study, we investigated the anti-tumor effect of several dual inhibitors of HDACs and GSK3B on ovarian cancer cell proliferation, migration, and invasion *in vitro* in human and mouse ovarian cancer cell lines and identified the Metavert analog APCS-540 as the most effective compound against ovarian cancer compared to other Metavert analogs. We also tested the anti-tumor activity of APCS-540 in cisplatin-resistant human ovarian cancer cells (A2780cis) and examined its effectiveness in overcoming chemoresistance. Subsequently, we examined the effect of APCS-540 *in vivo* on disease progression in a syngeneic ovarian cancer mouse model.

## 2. Materials and methods

This study was reviewed and approved by the Institutional Animal Care and Use Committee at Cedars-Sinai Medical Center, Los Angeles, California.

### 2.1. Cell lines

The syngeneic mouse ovarian cancer cell lines BR-Luc (*p53*−*/*−*, Brca1*−*/*−*, Myc, Myr-Akt*) and MOSE-HRas-Myc (*p53*−*/*−*, HRas, Myc*) were generated in the Orsulic Laboratory [10–12]. The ID8 cell line (*p53*−*/*−*, Brca2*−*/*−) was obtained from Dr. Ian McNeish (University of Glasgow, UK) [13]. The human ovarian cancer cell lines KURAMOCHI, OVCA420, and OVSAHO were kindly provided by Dr. Dennis Slamon (UCLA, USA). The Ishikawa (human endometrial adenocarcinoma) and SiHa (human cervical adenocarcinoma) cell lines were kindly provided by Dr. Kate Lawrenson (Cedars-Sinai Medical Center, USA). The ovarian cancer cell lines A2780 (cisplatin-sensitive) and A2780cis (cisplatin-resistant) were obtained from Fox Chase Cancer Center, PA, USA. None of the cell lines have been propagated in culture for more than 6 months. KURAMOCHI, OVCA420, OVSAHO, A2780 and A2780cis cells were cultured in RPMI 1640 media supplied with 10% fetal bovine serum (FBS). BR-Luc, ID8, Ishikawa, and SiHa cells were cultured in Dulbecco’s Modified Eagle Media (DMEM) supplied with 10% FBS.

### 2.2. Chemical compounds

Dual inhibitors of GSK3Β and HDACs (APCS-540, APCS-643, APCS-644) were synthesized by Albany Molecular Research Institute (AMRI, Albany, NY, USA) and Metavert was synthesized by Royal Pharma (Mumbai, India). Metavert is a trademark owned by Avenzoar Pharmaceuticals, Encinitas, CA, USA and is currently in the preclinical stage for treating pancreatic adenocarcinoma. The Pan-HDAC inhibitor suberoylanilide hydroxamic acid (SAHA) was purchased from Cayman (Ann Arbor, MI, USA). The GSK3B inhibitor Tideglusib was purchased from Sigma-Aldrich (St. Louis, MO, USA). D-luciferin for *in vivo* bioluminescence imaging was purchased from Goldbio (St. Louis, MO, USA).

### 2.3. Cancer cell survival assay

Survival of *in vitro* cultured cancer cells was assessed by crystal violet staining. Crystal violet is a triarylmethane dye that can bind to ribose type molecules such as DNA in the nuclei. It offers an efficient method for *in vitro* cell viability or cytotoxicity assessment under various conditions and drug screening. The amount of staining in the assay is directly proportional to the cell biomass and can be measured at 560–570 nm absorbance. Briefly, 5 × 10^3^/well cancer cells were seeded into 24-well plates and cultured overnight. The cells were subsequently treated with different concentrations (0.6–4.8 µM) of Metavert, APCS-540, APCS-643, APCS-644, SAHA, and Tideglusib and cultured for 72–96 h depending on the experimental setting. A cisplatin dose of 10 µM was used in the experiments with A2780 and A2780cis cell lines. All cell culture experiments were performed in quadruplicate, and the survival of treated cancer cells was compared to untreated controls.

### 2.4. Transwell cancer cell migration and invasion assay

The effect of APCS-540 at different concentrations (0.6 µM, 1.2 µM, and 2.4 µM) on migration and invasion ability of human and mouse ovarian cancer cell lines was evaluated using Boyden’s chamber (Transwell Assay) with Matrigel-coated Transwell inserts with a pore size of 8 µm. To create a chemotactic gradient, 10% FBS was added into the lower chamber media while cell media in the upper chamber were kept FBS-free. After 72 h of treatment, 50 × 10^3^ cancer cells were reseeded on the Transwell insert overnight at 37 °C and 5% CO_2_ to assess the effect of treatment on both migration and invasion. The Transwell inserts were later removed from the plate and residue culture media and cells that had not migrated from the upper side of the membranes were gently removed using a cotton-tipped applicator. Migrated cancer cells on the bottom side of the membranes were fixed with 10% formalin for 20 min, stained with crystal violet for 30–45 min, washed with PBS, and allowed to dry. Images of migrated cells were taken under an optical microscope and the cells were counted (average of 5 fields at 100× magnification).

### 2.5. RNA isolation and real-time quantitative PCR

Total RNA was extracted using Trizol (ThermoFisher, Canoga Park, CA, USA), and reverse transcription reaction was carried out using High Capacity Reverse Transcription Kit (Thermo Fisher, Canoga Park, CA, USA). Real-time quantitative PCR (RT-qPCR) was used for quantifying mRNA levels using the iTaq Universal SYBR Green Supermix (BioRad, Hercules, CA, USA) and BioRad cfx96 platform according to the manufacturer’s protocol. Gene expression levels were normalized to that of GAPDH. Primers were purchased from Integrated DNA technologies (IDT), Coralville, IA, USA. The sequences of primers used for RT-PCR were as follow: h-Oct4-F; GAGAATTTGTTCCTGCAGTGC, h-Oct4-R; GTTC CCAATTCCTTCCTTAGTG, h-CD133-F; GAGTCGGAAACTGGCAGATAGCA, h-CD133-R; ACGCCTTGTCCTTGGTAGTGTTG, h-Nanog-F; ACCTATGCC TGTGATTTGTGG, h-Nanog-R; AAGAGTAGAGGCTGGGGTAGG, h-YAP-F; TAGCCCTGCGTAGCCAGTTA, h-YAP-R; TCATGCTTAGTCCACTGTCTGT, h-GAPDH-F; CCAGGTGGTCTCCTCTGACTTCAACA, h-GAPDH-R; AGGGTC TCTCTCTTCCTCTTGTGCTC.

### 2.6. Western blot analysis

Cells were re-suspended in RIPA phosphorylation buffer (50 mM NaCl, 50 mM Tris/HCl pH 7.2, 1% deoxycholic acid, 1% Triton X-100, 0.1% SDS, 10 mM Na2HPO4 + NaH2PO4, 100 mM NaF, 2 mM Na_3_VO_4_, 80 µM glycerophosphate, 20% glycerol, 1 mM PMSF, 5 µg/mL each of pepstatin, leupeptin, chymostatin, antipain, and aprotinin). Lysates were then centrifuged for 15 min at 16,000 x g at 4 °C. Proteins in the supernatant were separated by SDS-PAGE and electrophoretically transferred to nitrocellulose or PVDF membranes. Non-specific binding was blocked for one hour with 5% bovine serum albumin or non-fat dry milk in Tris-buffered saline (4 mM Tris base, 100 mM NaCl, pH 7.5) containing 0.05% Tween 20. Membranes were incubated with primary antibody overnight at 4 °C, and then with peroxidase-conjugated secondary antibody for one hour. Blots were developed using SuperSignal Chemiluminescent Substrate (Pierce, Rockford, IL, USA).

### 2.7. Syngeneic mouse model of ovarian cancer and treatment protocol

For *in vivo* evaluation of the drug efficiency, 6-week-old female FVB mice (The Jackson Laboratory, Sacramento, CA, USA) were injected intraperitoneally (i.p.) with 1 × 10^6^ BR-Luc cells to simulate metastatic ovarian cancer dissemination in the mouse. One week after i.p. injection of cancer cells, all mice were imaged by luciferin bioluminescence to confirm the intraabdominal development of tumors. The mice were subsequently assigned to two groups with 9 mice in each group: 1) control (vehicle), and 2) treatment (APCS-540, 10 mg/kg). Mice were injected i.p. 3 times per week by a researcher who was blinded to the study groups.

### 2.8. In vivo bioluminescence imaging

To confirm tumor growth and metastasis to the abdominal organs after i.p. injection of cancer cells, mice were imaged using the IVIS Spectrum CT *In Vivo* Imaging System (Perkin Elmer Inc., Waltham, MA, USA). One week after cancer cell injection, the mice were anesthetized with 2% isoflurane and i.p. injected with D-luciferin solution (10 µL per gram of body weight; concentration of 15 mg/mL diluted in PBS). After D-luciferin injection, we waited 10 min to allow for luciferase activity; then, the mice were placed in the imaging chamber of the IVIS system. Images were obtained every 30 s until the peak value of bioluminescence was achieved.

### 2.9. IVIS image analysis

The obtained images were analyzed for bioluminescence signal intensity per mouse *via* the advanced *in vivo* imaging software, Living Image (Perkin Elmer Inc., Waltham, MA, USA). A standardized rectangular region of interest (ROI) was selected in each image to include the abdominopelvic cavity where tumor growth and metastasis were anticipated. The average photon count per second in the selected ROI was calculated and recorded for every image. Statistical analysis was performed to compare photon intensity between the treatment and control groups using GraphPad Prism software (GraphPad Software, CA, USA).

### 2.10. Animal survival assay

All mice were monitored for signs of morbidity at least three times weekly until they became moribund due to advanced metastatic disease. The survival duration for each mouse was calculated as the number of days from the initiation of treatment to euthanasia.

### 2.11. Statistical analysis

Collected data were analyzed using GraphPad Prism software. Comparisons were performed *via* Student *t*-test, Two-way ANOVA, or Fisher’s exact test. For animal survival data analysis, the long-rank (Mantel-Cox) was utilized. A *p*-value b.05 was considered statistically significant.

## 3. Results

### 3.1. Dual inhibition of GSK3B and HDACs decreases human and mouse cancer cell survival

**Fig. 1.**
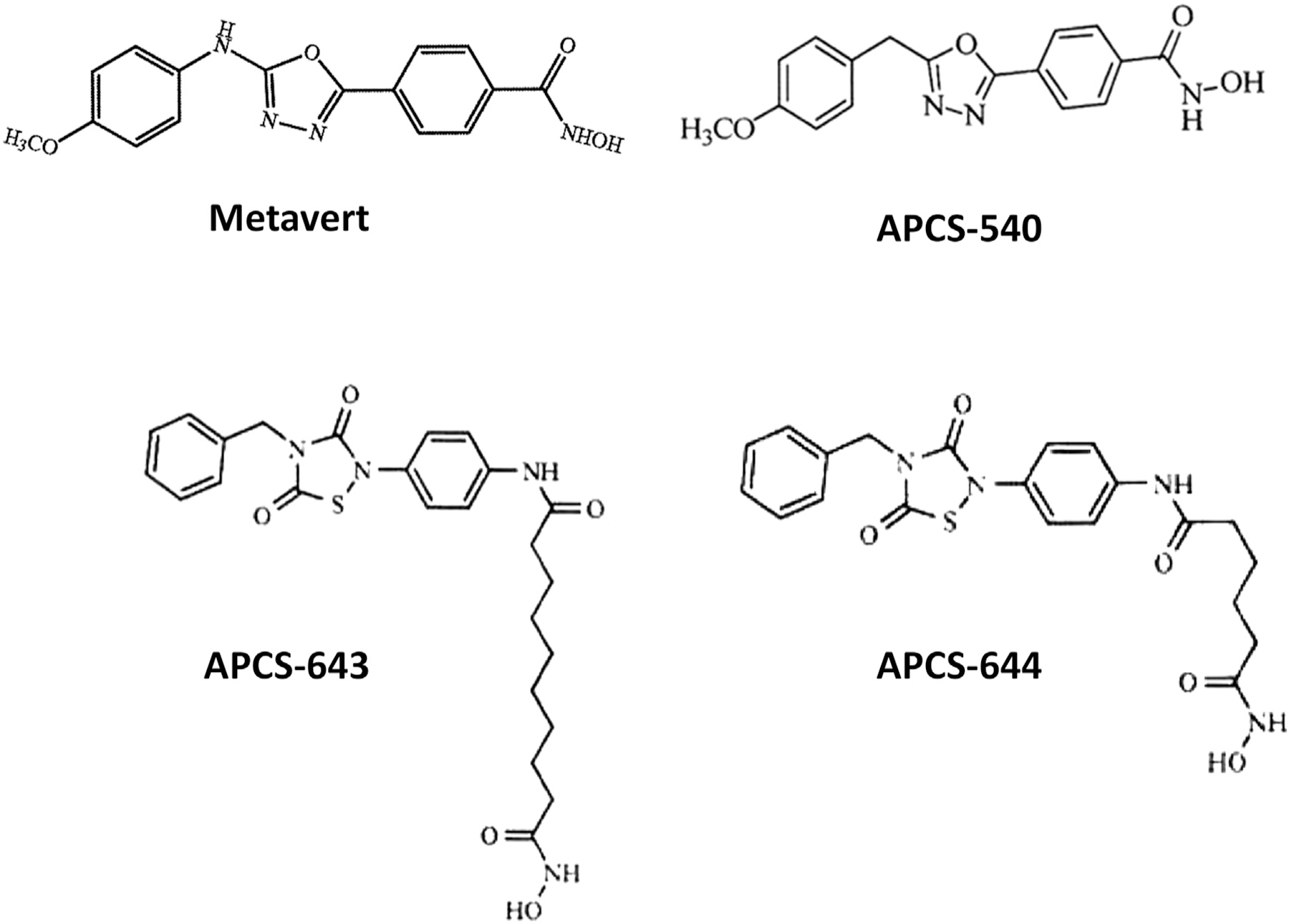
Chemical structures of newly-developed dual GSK3B and HDACs inhibitors.

**Fig. 2.**
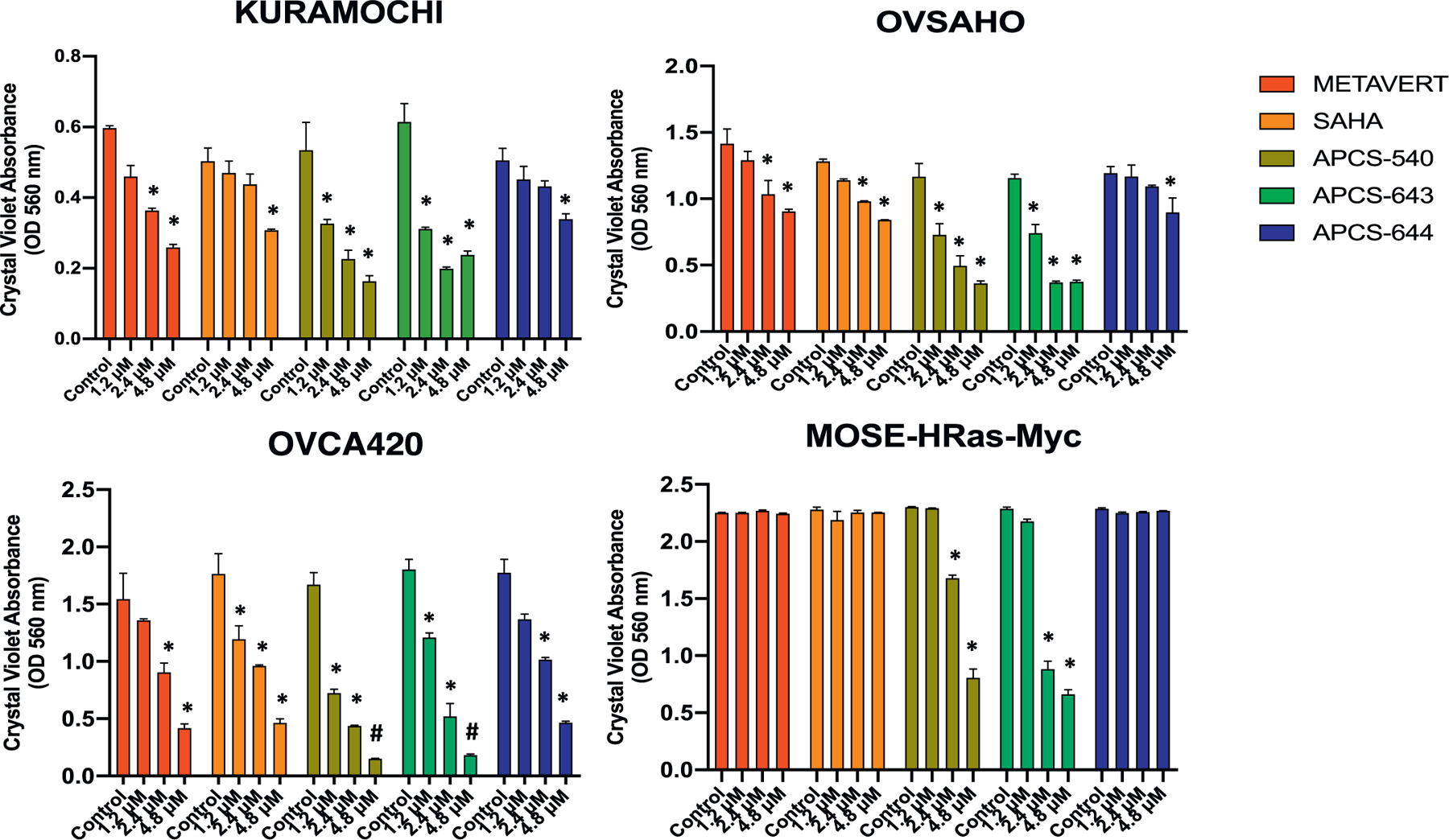
The effects of dual GSK3B and HDACs inhibitor analogs on cancer cell survival compared to SAHA in three human (KURAMOCHI, OVCA420, OVSAHO) and one mouse (MOSE-HRas-Myc) ovarian cancer cell lines. * represents *p* < .05, and # represents *p* < .001 compared to control.

We first examined whether simultaneous inhibition of GSK3B and HDACs is more effective than inhibition of HDAC alone in reducing the survival of ovarian cancer cells *in vitro.* We designed and synthesized four dual inhibitors (Metavert, APCS-540, APCS-643, APCS-644), which were tested in comparison to the HDAC inhibitor SAHA in human (KURAMOCHI, OVSAHO, OVCA420) and mouse (MOSE-HRas-Myc) cell lines at concentrations of 1.2 µM, 2.4 µM, and 4.8 µM *vs.* control. The chemical structures of Metavert and its analogs are shown in Fig. 1. All dual inhibitor analogs significantly reduced cancer cell survival in a dose-dependent manner in all human cancer cell lines with the most potent effect observed in OVCA420 cells (Fig. 2). At a concentration of 4.8 µM, N95% of cancer cells were killed by APCS-540 and APCS-643 compared to control (*p* < .001 and p < .001, respectively). Even at 1.2 µM concentration, APCS-540 reduced cancer cell survival significantly by 40% to 60% compared to controls in the KURAMOCHI, OVSAHO, and OVCA420 cell lines. SAHA showed no significant effect at 1.2 µM concentration except in the OVCA420 cell line (*p* = .02 at 1.2 µM *vs.* control). The anti-tumor effect was even more evident in the cancer cells treated with APCS-540 and APCS-643. When we tested these agents in the MOSE-HRas-Myc mouse ovarian cancer cells, which have a highly aggressive proliferative behavior, only APCS-540 and APCS-643 (*p* = .03 at 2.4 µM *vs.* control, and p = .02 at 4.8 µM *vs.* control) demonstrated significant anti-tumor effects compared to Metavert, APCS-644, and SAHA (Fig. 2). Comparison of the effects of APCS-540 and Metavert on cancer cell survival showed that the effect of APCS-540 is significantly bigger than the effect of Metavert in most of the doses tested and in the 4 cell lines (Fig. 2).

**Fig. 3.**
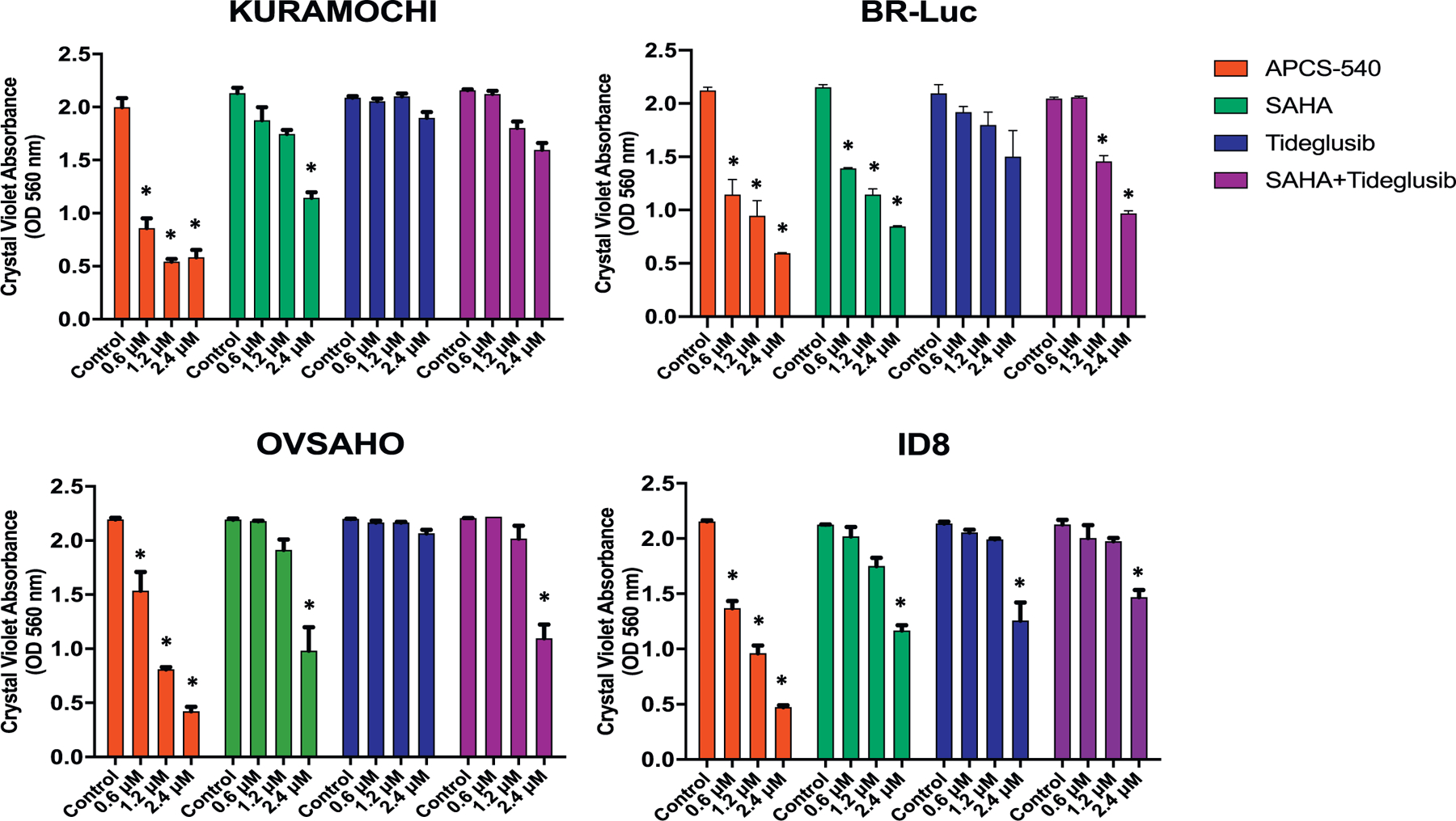
The effects of GSK3B (Tideglusib) and HDAC (SAHA) inhibitors alone and in combination on cancer cell survival compared to APCS-540 in two human (KURAMOCHI, OVSAHO) and two mouse (BR-Luc, ID8) ovarian cancer cell lines. * represents *p* < .05.

After these initial experiments, which showed that analog APCS-540 possessed the highest anti-tumor activity, we investigated its effect on cancer cell survival in comparison to Tideglusib (GSK3Β inhibitor) and SAHA (HDAC inhibitor) alone and in combination. APCS-540 reduced the viability of human and mouse ovarian cancer cells to a greater extent than Tideglusib and SAHA alone or in combination (Fig. 3). These results suggest that dual targeting of GSK3B and HDACs has better anti-tumor activity than the combination of agents singly targeting these oncogenic pathways.

Recent studies have shown that inhibition of GSK3B and HDACs induces anti-tumor effects in endometrial and cervical cancer [14–17]. To provide preliminary data on the anti-tumor potential of APCS-540 in these gynecologic malignancies, we treated human cervical (SiHa) and endometrial (Ishikawa) cancer cell lines with different concentrations of APCS-540. The results showed that 1.2 µM concentration of APCS-540 significantly reduced survival in both cancer cell lines (*p* = .02 and *p* = .03, respectively). Combination treatment with SAHA and Tideglusib also effectively induced cell death in SiHa and Ishikawa cancer cell lines at 2.4 µM concentration (Supplementary Fig. S1). These promising results suggest that dual inhibition of GSK3Β and HDACs may be effective in a wider spectrum of gynecologic tumors.

### 3.2. Ovarian cancer cell migration and invasion are significantly reduced with APCS-540 treatment

**Fig. 4.**
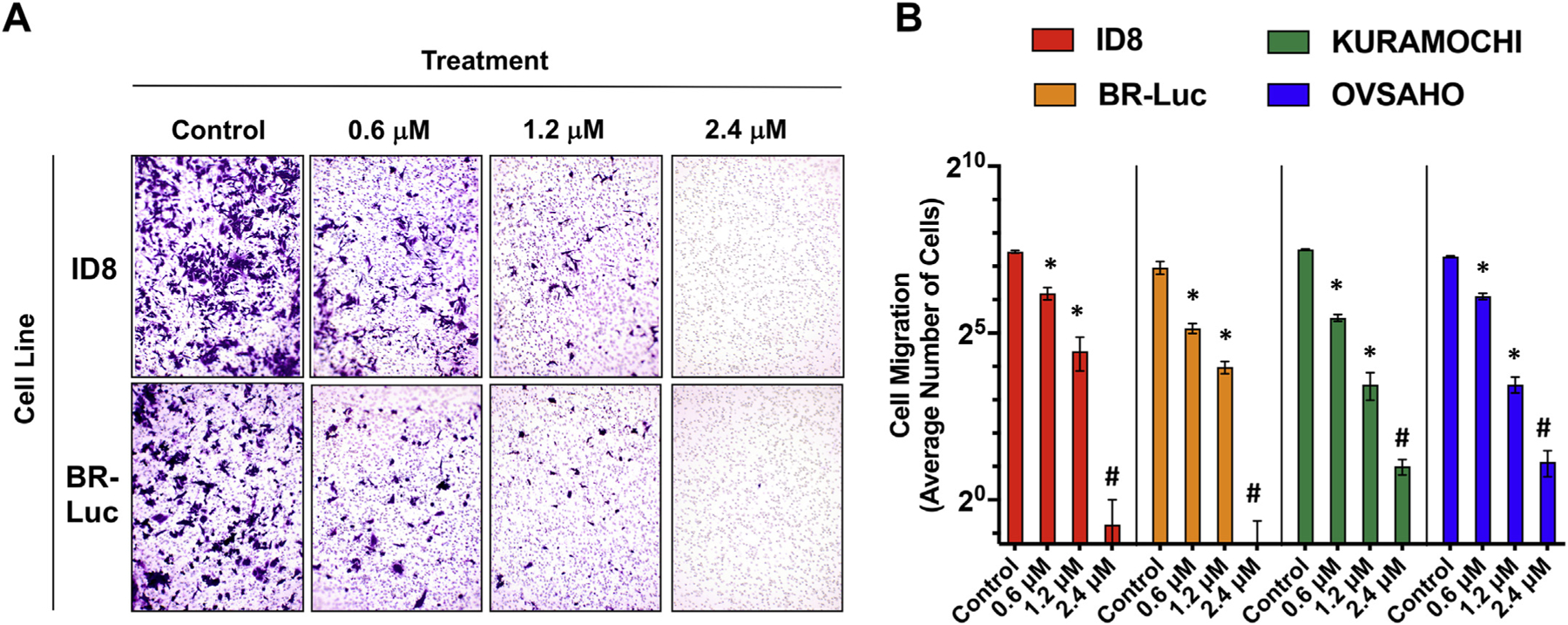
Cancer cell migration and invasion assay results. A) Representative Transwell migration and invasion assay images (100× magnification) from ID8 and BR-Luc cancer cells treated with different concentrations of APCS-540. Cancer cell migration and invasion abilities of both ID8 and BR-Luc cells were significantly reduced with 0.6 µM of APCS-540, and completely inhibited at 2.4 µM. B) Average number of migrated cancer cells treated with APCS-540 concentrations of 0.6 µM, 1.2 µM, and 2.4 µM compared to control in mouse (ID8, BR-Luc) and human (KURAMOCHI, OVSAHO) ovarian cancer cells. Average number of cells is given at log2 scale. * represents p < .05, and # represents p < .001 compared to control.

After confirming the cytotoxic effect of APCS-540 in cancer cells, we examined its effect on cancer cell migration and invasion, which are the essential determinants of the metastatic capacity of cancer cells. Using a Matrigel-coated Transwell assay, we found that APCS-540 significantly reduced the ability of the highly invasive mouse ovarian cancer cell lines ID8 and BR-Luc to migrate and invade through Matrigel at 0.6 µM and 1.2 µM and almost completely inhibited migration and invasion at 2.4 µM concentration (Fig. 4A, B). Similar results were observed in human ovarian cancer cell lines KURAMOCHI and OVSAHO (Fig. 4B). These results showed that APCS-540 not only reduced cancer cell viability but also inhibited the metastatic capacity, which is crucial for an effective treatment strategy.

### 3.3. APCS-540 reduces survival of cisplatin-resistant ovarian cancer cells and reverses chemo-resistance

**Fig. 5.**
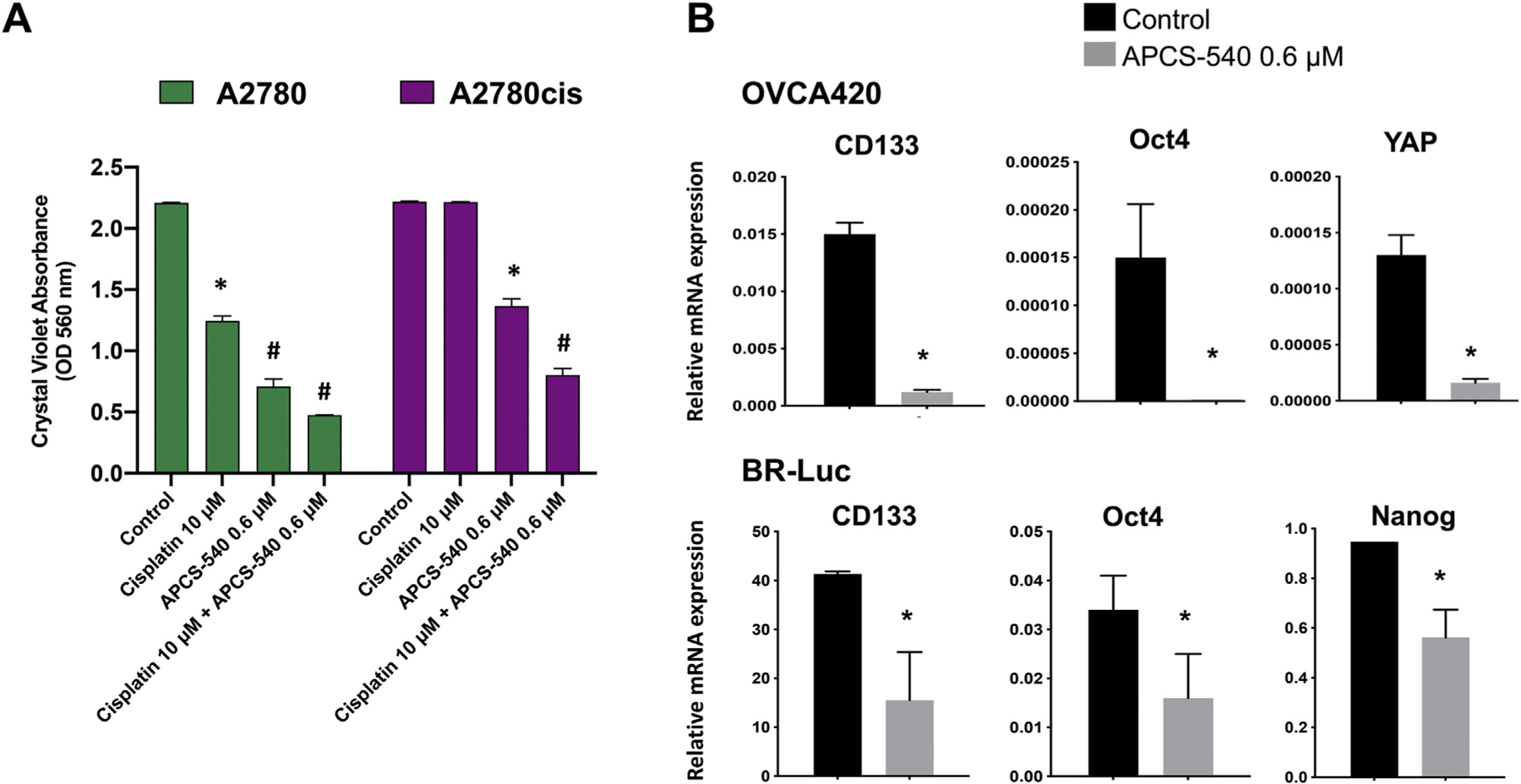
A) The effect of APCS-540 on cisplatin-sensitive (A2780) and cisplatin-resistant (A2780cis) human ovarian cancer cells. B) APCS-540 (0.6 µM) decreased relative mRNA expression levels of cancer stemness markers in human (OVCA420) and mouse (BR-Luc) ovarian cancer cell lines cultured for 48 h. Relative mRNA expression level was determined based on the housekeeping gene mRNA expression level. * represents p < .05, and # represents p < .001 compared to control.

Because chemoresistance is one of the major factors in tumor recurrence and worse prognosis, we aimed to evaluate the anti-tumor activity of APCS-540 in the cisplatin-sensitive human ovarian cancer cell line A2780 and its cisplatin-resistant derivative A2780cis alone or in combination with cisplatin (10 µM). Western blot analysis of acetylated histone 3 lysine 9 (Ac-H3K9) and beta-catenin, which is the direct target of GSK3B, protein levels re-probed for glyceraldehyde 3-phosphate dehydrogenase (GAPDH) showed that treatment with 0.6 µM APCS-540 significantly inhibited HDAC and GSK3B pathways in both A2780 and A2780cis cancer cells compared to control (Supplementary Fig. S2A). Remarkably, APCS-540 reduced cancer cell viability in both A2780 and A2780cis cancer cells at a low concentration of 0.6 µM (*p* < .001) (Fig. 5A). When combined with cisplatin, APCS-540 showed an even greater cytotoxic effect in both cell lines. These findings indicate that APCS-540 can reverse chemoresistance and synergistically improve chemotherapy response in ovarian cancer cells.

### 3.4. APCS-540 reduces expression of stem cell markers in ovarian cancer cells

Additionally, we examined whether APCS-540 had any effect on cancer stemness, which is a critical factor mediating drug resistance. RT-qPCR analysis of stemness markers CD133, Oct4, and Nanog and pro-tumorigenic marker YAP in OVCA420 and BR-Luc cancer cells treated with 0.6 µM APCS-540 showed a significant decrease in expression levels of these markers (Fig. 5B). Similarly, significant results were observed with Oct4 and Nanog in A2780 and A2780cis cancer cells (Supplementary Fig. S2B). These results, together with the reversal of chemoresistance, suggest APCS-540 is a promising anti-tumor agent for the treatment of ovarian cancer.

### 3.5. Treatment with APCS-540 increases survival in mice with ovarian cancer

**Fig. 6.**
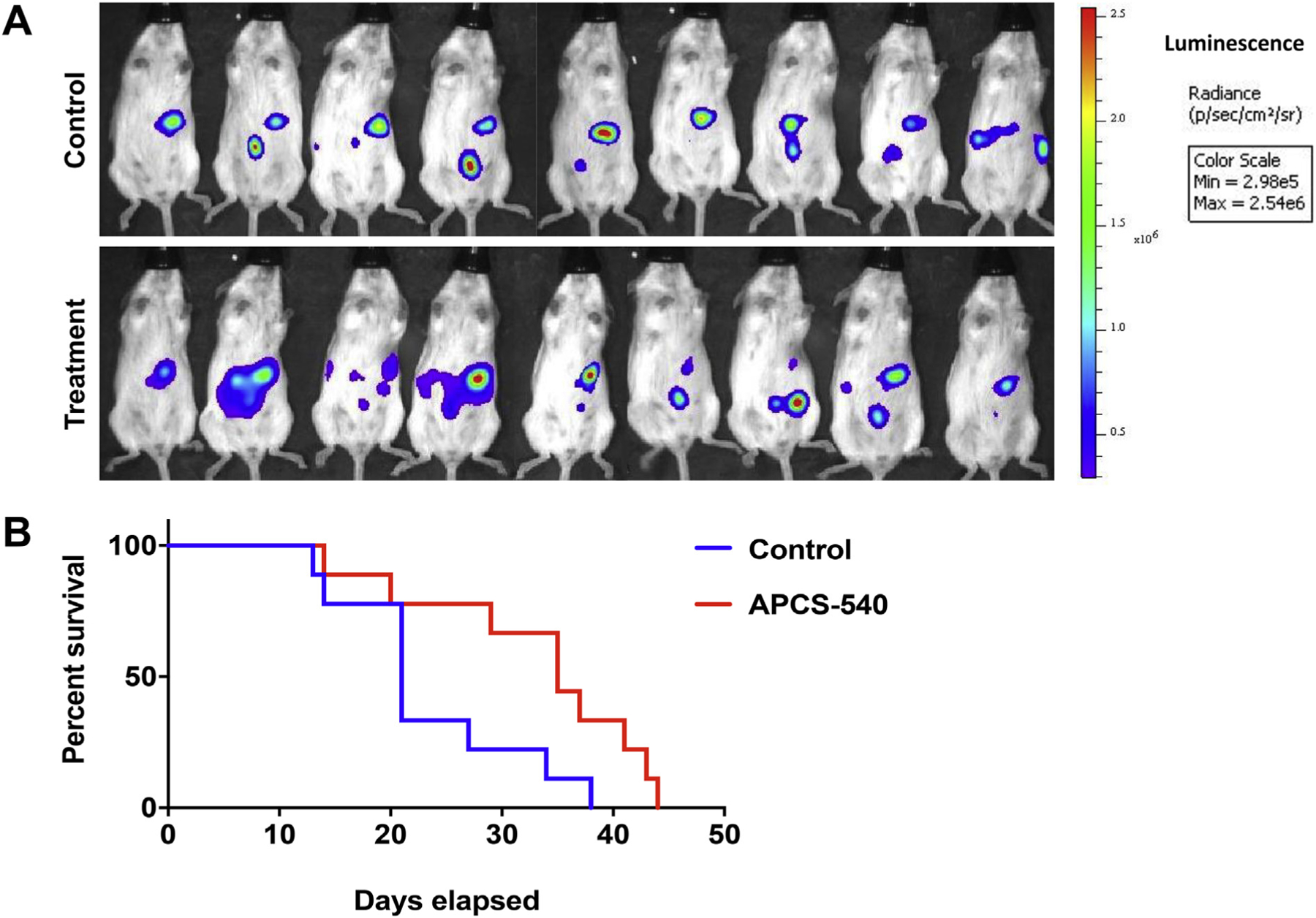
A) Baseline (pre-treatment) IVIS imaging results of mice i.p. injected with BR-Luc cells and assigned to the control (*n* = 9) and treatment (n = 9) groups. Imaging confirmed that all mice in both groups harbor intra-abdominal growing tumors. No statistically significant difference was observed at baseline between two groups. B) Survival assay results for mice treated with APCS-540 *vs.* control. Treatment with APCS-540 significantly increased survival in tumor-bearing mice by 66%.

To test whether APCS-540 can prevent ovarian cancer progression and metastasis, we used an immunocompetent syngeneic ovarian cancer mouse model, which successfully simulates human metastatic ovarian cancer [11]. BR-Luc mouse ovarian cancer cells were i.p. injected into twenty 6-week-old female FVB mice. One week after cancer cell injection, mice were imaged *via* IVIS bioluminescence imaging to confirm intraabdominal tumor growth. IVIS imaging showed 18 mice with growing tumors and 2 mice with no measurable luciferin activity. Hence, the 2 mice without tumors were excluded from the experiment. Mice with confirmed tumor growth were assigned randomly to treatment (*n* = 9) and control (n = 9) groups (Fig. 6A). Mice in the treatment group were i.p. injected three times a week with APCS-540 (10 mg/kg). The median survival in the control group was 21 days, whereas the median survival in the treatment group was 35 days (Fig. 6B). This result showed a 66% increase in survival when mice are treated with APCS-540 (*p* = .03).

Furthermore, during abdominal dissection of euthanized mice, we observed more disseminated disease in the control group with multiple tumor lesions in the peritoneum, omentum, intestines, and stomach. At the same time, less than half of the APCS-540 treated mice had tumors growing on the peritoneal surfaces of all of these organs, indicating a significant decrease in tumor metastasis. This observation was in accordance with our *in vitro* results that showed reduced migration and invasion capability of BR-Luc cells treated with APCS-540.

## 4. Discussion

Despite remarkable advances in the treatment of several hematological cancers and solid tumors, there has been no significant improvement in the treatment of ovarian cancer, which remains the deadliest gynecologic malignancy in the United States [1]. Standard of care treatment in ovarian cancer is debulking surgery combined with platinum/taxane-based chemotherapy. However, despite these aggressive treatments, tumor recurrence occurs in approximately 80% of patients. In the last decade, an increasing number of studies on cancer evolution have shown that evolving tumor clones that acquire chemoresistance are the primary cause of tumor recurrence [18,19]. Therefore, overcoming chemoresistance is a crucial challenge in developing an effective treatment for ovarian cancer.

Commonly used chemotherapeutic agents in ovarian cancer primarily target genomic integrity (platinum analogs) or cell division (taxanes). However, cancer cells can undergo extensive molecular changes at genetic, epigenetic, and metabolic activity levels as a response to different cellular stresses, such as chemotoxicity, and adapt to the environment by utilizing alternate cellular pathways, which results in increased cellular plasticity and chemoresistance [20]. A promising approach to overcome chemoresistance is simultaneous targeting of multiple key oncogenic pathways. Because the development of resistance to a single target is almost universal, combinations of drugs targeting different cellular pathways, such as epigenetic and metabolic pathways, may yield more effective anti-cancer therapies. A combination of multiple agents may provide an additive effect in killing cancer cells; however, it can also cause potential side effects due to drug toxicity and drug-drug interactions. Thus, a multitargeted single drug can provide superior pharmacokinetics and pharmacodynamics with lower side effects. Based on this knowledge, we focused on two oncogenic pathways, metabolic (GSK3B) and epigenetic (HDAC), and developed a novel drug that targets these two pathways simultaneously. To our knowledge, this is the first report of using a dual GSK3B/HDAC inhibitor for the treatment of ovarian cancer.

GSK3B is a serine/threonine kinase and a glycogen metabolism enzyme involved in several NF-κB signaling pathways. It has been proposed as a new therapeutic target for various cancers due to its essential roles in tumor proliferation and resistance to apoptosis [6]. Data from a recent study showed that inhibition of GSK3B using a small molecule (9ING41) significantly induced apoptosis in SKOV3 and OVCA432 ovarian cancer cell lines and reduced *in vivo* tumor growth in mice [7]. However, it should be noted that inhibiting GSK3B alone can also play a pro-cancer role, promoting epithelial to mesenchymal transition (EMT; a measure of metastatic potential) and cancer stemness (a measure of resistance to chemotherapy) [21,22]. Hence, single inhibition of GSK3B is an imperfect method that needs a complementary method to overcome these adverse effects.

Histone deacetylases (HDACs) are epigenetic modulators that play a crucial role in carcinogenesis by promoting cancer stemness and EMT. HDACs were shown to interact with EMT transcription factors leading to the promotion of EMT in cancer cells [23]. Successful results using HDAC inhibitors ultimately led to clinical trials and FDA-approved treatments for various cancers, such as advanced lymphoma and metastatic cancers [24–26]. In a recent study, HDAC inhibitors were reported to be effective in killing ovarian cancer cells and reducing tumor growth by suppressing oncogene PAX8 expression in a mouse model [8]. Considering this background, we hypothesized that simultaneously inhibiting GSK3B and HDACs would prevent cancer cell growth while also inhibiting EMT and its effect on metastasis and chemoresistance.

In this study, we investigated the effect of first-in-class dual inhibitor agents of GSK3B and HDACs for the treatment of ovarian cancer. Our results showed that dual inhibition of GSK3B and HDACs significantly reduced cancer cell survival both in human and mouse ovarian cancer cell lines. Among the developed dual inhibitor agents, APCS-540 showed the best anti-tumor activity. *In vitro* experiments using both human and mouse ovarian cancer cell lines also demonstrated that APCS-540 significantly reduced cancer cell migration and invasion, demonstrating its effectiveness in inhibiting the metastasis capacity. More importantly, APCS-540 displayed significant cytotoxic activity in cisplatin-resistant ovarian cancer cells, and remarkably reversed chemoresistance when combined with cisplatin. In an immunocompetent syngeneic mouse model, APCS-540 significantly improved survival by 66%. Additionally, our preliminary *in vitro* results in cervical and endometrial adenocarcinoma cell lines showed that APCS-540 may be effective in a wider spectrum of gynecologic malignancies.

In conclusion, we showed significant effectiveness of simultaneous targeting of the cancer epigenome and metabolism in preclinical cancer models. Our results suggest APCS-540 as a promising therapeutic drug for ovarian cancer, including the platinum-resistant disease.

## Supplementary Material

1

2

## HIGHLIGHTS

- Dual inhibition of GSK3B and HDACs reduces cancer cell survival in both human and mouse ovarian cancer cell lines.
- GSK3B/HDACs dual inhibitor APCS-540 reduces ovarian cancer cell survival, migration and invasion.
- APCS-540 is cytotoxic to cisplatin-resistant human ovarian cancer cells and re-sensitizes cancer cells to cisplatin.
- APCS-540 reduces cancer stemness markers CD133, Oct4, Nanog and YAP in ovarian cancer cells.
- APCS-540 improves survival in a syngeneic ovarian cancer mouse model.
