# Supplementary figures and images for: Dual targeting of GSK3B and HDACs reduces tumor growth and improves survival in an ovarian cancer mouse model
Source: Gynecol Oncol. Author manuscript; Available in PMC 2020 Dec 28. (PMC7769125; doi:10.1016/j.ygyno.2020.07.005)

A

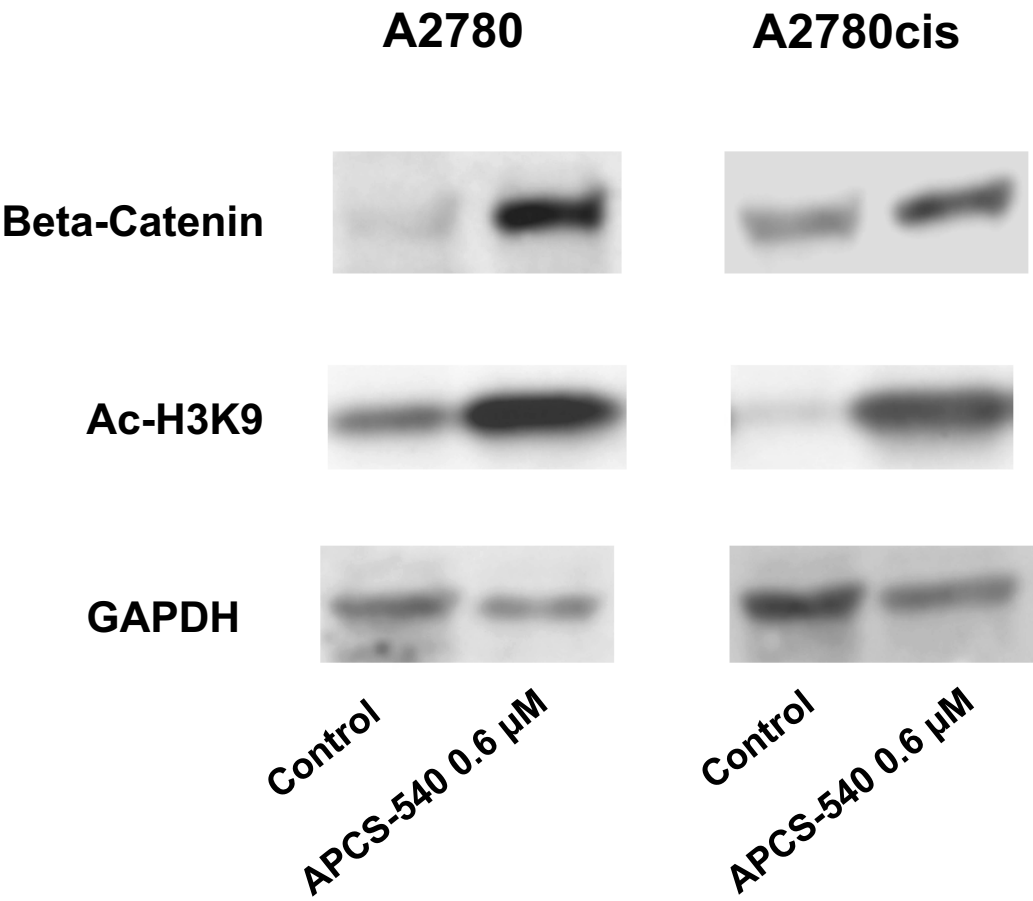

B

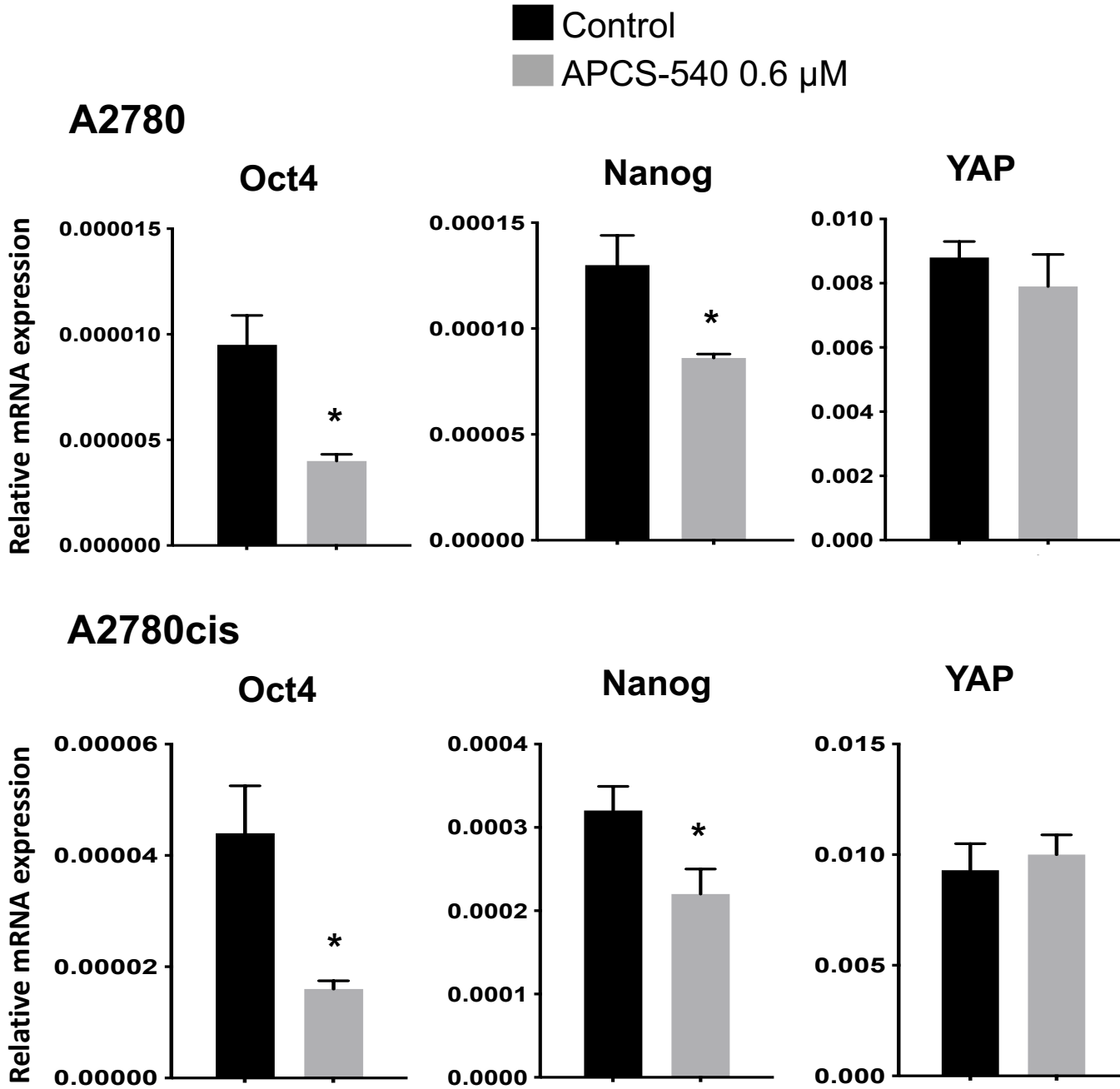

Supplement: 1 [file NIHMS1611620-supplement-1.pdf]

Supplementary Figure S1

SiHa

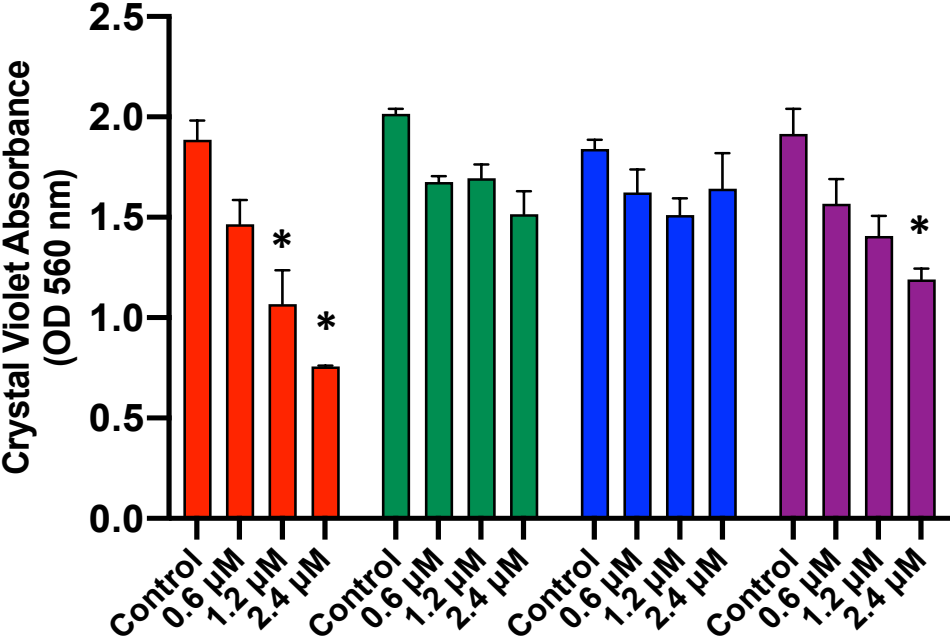

Ishikawa

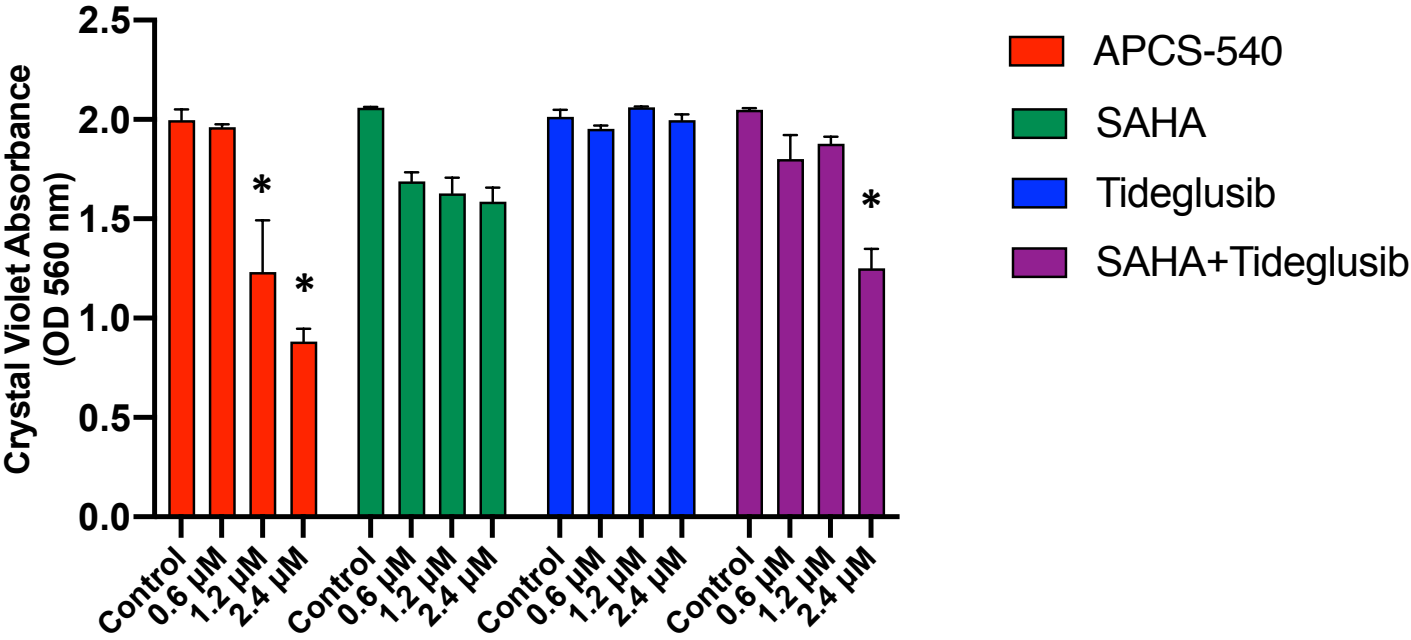

Supplement: 2 [file NIHMS1611620-supplement-2.pdf]
